# New mechanistic insight: neural cell death in diabetes-linked depression

**Published:** 2014-06

Diabetes, a prevalent metabolic disorder worldwide, can co-occur with depression. Depression is a debilitating mental health condition that often requires treatment with antidepressant medication. Although this approach is usually effective in managing clinically diagnosed depression, depression that is comorbid with diabetes responds poorly to currently available antidepressants. This has fuelled the idea that the underlying pathophysiology of diabetes-associated depression might be fundamentally different, emphasising the need for increased research efforts in this area. In this study, Guyu Ho and co-authors demonstrate that an established mouse model of diabetes displays anxiety- and depression-like behaviours, supporting the comorbidity of depression and diabetes. By applying immunohistochemical and biochemical approaches in areas of the brain that are known to govern mood control, they provide evidence that neural cell death occurs in association with the depressive phenotype of diabetic mice. Furthermore, they show that hyperglycaemia-induced mitochondrial dysfunction could play a role in driving apoptosis in brain cells. These findings suggest that, in contrast with standalone depression, neural cell death could contribute to depression that occurs with diabetes. This could have implications for the development of new neural-protective therapies to treat depression in individuals with diabetes. **Page 723**

